# Correspondence on “Mortality Pattern of *Poecilus cupreus* Beetles after Repeated Topical Exposure to Insecticide—Stochastic Death or Individual Tolerance?”

**DOI:** 10.1021/acs.est.4c03056

**Published:** 2024-06-06

**Authors:** Roman Ashauer

**Affiliations:** † Syngenta Crop Protection AG, 4058 Basel, Switzerland; ‡ Department of Environment and Geography, University of York, York YO10 5NG, U.K.

In their recent publication “Mortality Pattern of *Poecilus cupreus* Beetles after Repeated Topical Exposure to Insecticide—Stochastic Death or Individual Tolerance?”, Sowa et al.^1^ investigate whether stochastic death (SD) or individual tolerance (IT) can better explain their data. The experimental design is similar to that of the hypothetical experiment outlined and explained in more detail elsewhere.^2^ Briefly, groups of organisms exposed repeatedly to the same dose of a toxicant, with a recovery period between exposures, would exhibit different mortality patterns under the assumption of SD or IT. To understand and interpret such data, it is important to also consider toxicokinetics (TK) and toxicodynamics (TD),^3^ e.g., because the same data can be explained with either SD or IT but IT generally results in slower elimination and/or slower damage repair compared to SD to explain the same data.^2^ This is why it is good that Sowa et al. applied the toxicokinetic–toxicodynamic model GUTS^4^ to their data, specifically the reduced versions GUTS-RED-SD and GUTS-RED-IT,^5,6^ which are implemented in the user-friendly, open-source software openGUTS (https://openguts.info/). However, there is one error in the study by Sowa et al. that needs to be corrected, and I also want to take this opportunity to comment on some other aspects.

Sowa et al. exposed groups of carabid beetles to three subsequent dosing events by applying a 1 μL droplet of toxicant onto each individual’s scutellum on days 0, 29, and 66. Their chosen study design, which in addition to GUTS analysis also includes comparison of survival curves using Kaplan–Meier analysis, allows investigation of SD versus IT if all of the organisms received the same dose at each of the three exposure events. This is an important condition of the intended study design and a prerequisite for the data analysis strategy followed by Sowa et al. Unfortunately, there appear to be two problems related to this (first and last point below).

First, Sowa et al. implemented the exposures in openGUTS with the following time series: time (0, 0.5, ..., 28, 29, 29.5, ..., 65, 66, 66.5, ...) and exposure (30, 0, ..., 0, 30, 0, ..., 0, 30, 0, ...). The problem here is that openGUTS linearly interpolates the exposure from one time point to the next. This means the second exposure event starts already on day 28 and the third exposure event starts already on day 65 (see the “Exposure data entry error correction” figure). This does not correspond to the experiment carried out by Sowa et al., in which these exposures start on days 29 and 66, respectively. Importantly, this error does not occur on the first exposure event, which starts on day 0. Consequently, the exposure implemented in openGUTS for the second and third exposures is 3 times that of the first exposure event. This error can easily be corrected by using the following time series: time (0, 0.5, ..., 29, 29, 29.5, ..., 66, 66, 66.5, ...) and exposure (30, 0, ..., 0, 30, 0, ..., 0, 30, 0). Immediate changes from one exposure situation to the next can be entered into openGUTS by having two entries for a time point (see openGUTS example file “propiconazole_pulsed_renewals.txt” and page 12 of the openGUTS user manual, https://openguts.info/download.html). I attach corrected openGUTS files and openGUTS reports as Supporting Information.

Correcting for this error changes the results and at least some of the conclusions of the study by Sowa et al. The corrected openGUTS model fit results in different model parameter values and a different fit to the survival curves. For example, contrary to the original study, all goodness of fit statistics (NSE, NRMSE, and AIC) indicate that the GUTS-RED-SD model explains the data better than the GUTS-RED-IT model and that this is the case for beetles originating from the meadows as well as for beetles originating from oilseed rape fields (see Table 1). I do not view the differences in the goodness of fit between GUTS-RED-SD and GUTS-RED-IT in this study as being large enough to justify excluding the IT model, and both meet the acceptability criteria set by EFSA.^7^ Instead, I would use and interpret both SD and IT, but because Sowa et al. based their conclusions partly on the goodness of fit indicators, it is important to correct the record on that matter.

**Table 1:** Comparison of the SD and IT openGUTS Model Fits (corrected version of Table 2 of ref (1)) (DRT95, organism depuration/repair time) (95% confidence intervals)

model parameters and goodness of fit statistics	meadows, SD	meadows, IT	OSR, SD	OSR, IT
*k*_d_	1.754 (0.2117–4.129)	0.01719 (0.005367–0.02925)	0.3909 (0.1994–9.689)	0.004696 (0.0005637^a^–0.02241)
*m*_w_	2.645 (2.323 × 10^–5^^a^ to 4.713)	0.4749 (0.1702–0.8132)	2.519 (2.323 × 10^–5^^a^ to 6.625)	0.1601 (0.01708–0.4353)
*b*_w_	0.07028 (0.02677–68.44)		13.87 (0.01247–199.2)	
*F*_s_		20 (6.184–20^a^)		8.867 (2.742–20^a^)
DRT95 (days)	1.708 (0.7255–14.15)	174.3 (102.4–558.2)	7.664 (0.3092–15.02)	637.9 (133.7–5314)
NSE, r-square	0.8143	0.6681	0.9085	0.826
NRMSE (%)	24.12	32.33	12.37	17.14
AIC	987.9	1075.99	974.65	996.26

a The edge of the 95% parameter CI has run into a boundary.

Also, the mortality of the beetles after the first exposure is now captured better by the model (see corrected Figure 6). What Sowa et al. described as “Both the IT and SD models failed to accurately predict the high mortality of the beetles from meadows in the first 24 h after the first pesticide dose, seriously underestimating it.” was an artifact caused by the modeling error previously mentioned. Similarly, statements like “This was likely due to the lack of high mortality immediately after the first treatment, which GUTS was unable to accurately model for beetles from meadows” are no longer supported by the evidence. Other observations also need to be corrected, e.g., “The overall IT-driven mortality of beetles from meadow habitats exposed to three consecutive doses of insecticide was also indicated by the slightly better fit of the GUTS-RED-IT than the GUTS-RED-SD model”, because the data now show the opposite.

Second, because Sowa et al. make a case for IT on the basis that IT “follows the principles of natural selection” it is important to also consider the systematically slower damage repair under the IT model.^2^ The dominant rate constant (*k*_d_) can be converted into the damage repair time (DRT95) in GUTS-RED models (see Table 1), which are much longer for IT (hence, damage is building up in subsequent exposures) and longer for beetles from oilseed rape landscapes. How is that explained by selection?

Third, Sowa et al. used the survival data from treatments A-2 and A-3 as control data in the GUTS modeling to estimate the background mortality rate. However, I deleted those data from the control survival time series because these organisms had been exposed to the toxicant (all part of treatment P-1) and it cannot be assumed that they have had enough time for complete depuration and damage repair from that first exposure within 4 weeks. In fact, the GUTS-RED-IT model fits suggest that the depuration/repair time (DRT95) is much longer than 4 weeks (174 and 638 days for meadow beetles and oilseed rape beetles, respectively), and therefore, the mortality in those individuals could still be due to the initial toxicant exposure (P-1 treatment). However, this correction does not result in relevant changes to any of the model parameters or the model fits, because the background hazard rates are 0.006915 and 0.00441 day^–1^ for the meadows and oilseed rape beetles, respectively, in the original publication and 0.007008 and 0.004725 day^–1^, respectively, after correction, i.e., practically the same (these openGUTS files are also provided as Supporting Information). The GUTS analysis could be even further improved by using the observations of mortality recorded 2, 4, 6, 8, 10, and 12 h immediately after the exposures (better temporal resolution during the period in which a majority of deaths occurred) and by creating additional treatment groups in the model to match all of the experimental groups shown in Figure 2 of ref (1); however, I did not make those changes.

Finally, the beetles from the test population vary in body size and therefore in received dose. That the mass of the beetles used by Sowa et al. can vary substantially can be seen from Figure 5 of their previous study,^8^ which shows dry masses for parent generation beetles ranging from ∼0.015 to 0.055 g, with an approximate median value of 0.03 g and 25th and 75th percentiles of ∼0.024 and ∼0.037 g, respectively. This means that their smaller test organisms would have received ∼3 times the dose of the larger individuals if expressed per body mass and assuming similar body size variability in this study. Therefore, the stronger mortality after the first exposure event could be explained by the different doses received, where smaller individuals would have died first because of the greater dose that they received. Thus, IT is not the only explanation for the mortality in beetles from the meadows, although the lack of such greater mortality after the first exposure in beetles from the oilseed rape fields remains to be explained. As no data are provided on the size distributions in the different treatments and at the subsequent exposure events, it is impossible to know how much variability there was in each beetle’s dose. This makes conclusions about SD versus IT from this data set difficult.

The concepts of SD and IT have a long history, going back a century (see Chapter 1.5 of the GUTS book^3^), and GUTS provides a unifying mathematical framework for both^4^ (see also Chapters 2 and 3 of the GUTS book^3^). Fitting GUTS to a survival data set, and comparing the goodness of fit, is one way to test whether the hypotheses of SD or IT better explain the data. The body of literature on GUTS model applications shows that we can currently not select IT or SD as better performing (e.g., over 100 data sets^2,5,9−12^). Generally, both SD and IT explain the data well, and sometimes one or the other fits better, but not consistently across data sets. Comparisons of the concepts of SD and IT require considering much more nuance and more aspects than what is presented by Sowa et al. For example, see Chapter 1.4.2 of the GUTS book for a summary of the debate on this topic. It is only due to parameter identifiability considerations that typically the SD or IT variants are used separately; the actual GUTS unifies both concepts within one model. The GUTS TKTD framework is also helpful in other ways. For example, the full GUTS model treats TK and TD separately, and this allows GUTS-IT to model the phenomenon of delayed toxicity, which the reduced GUTS-RED-IT model cannot do, but which is straightforward in all SD variants.^13^

Perhaps most importantly, the question of how toxicant exposure results in selection and therefore evolution should be broadened beyond the threshold parameter. GUTS offers several parameters that can vary among individuals, such as the uptake and elimination rate constants to capture toxicokinetic differences as well as the damage accrual and damage repair rate constants to represent toxicodynamic differences. These model parameters are present in the SD and IT variants and can be viewed as phenotypic traits susceptible to selection pressure. Future analyses could, for example, use GUTS data analysis to investigate whether TK traits or TD traits are under more selective pressure.

## Supporting Information Available

Figure illustrating the exposure error in the original study (PDF)
Figure comparing the original and corrected GUTS-RED-SD model fit to the survival of the exposed meadow beetles (PDF)
Corrected openGUTS reports (PDF)
Corrected openGUTS reports (PDF)
Corrected openGUTS files (ZIP)
