# Supplementary figures and images for: Correspondence on “Mortality Pattern of Poecilus cupreus Beetles after Repeated Topical Exposure to Insecticide—Stochastic Death or Individual Tolerance?”
Source: Environ Sci Technol. 2024 Jun 6;58(24):10874–6. doi: 10.1021/acs.est.4c03056 (PMC11191583; doi:10.1021/acs.est.4c03056)

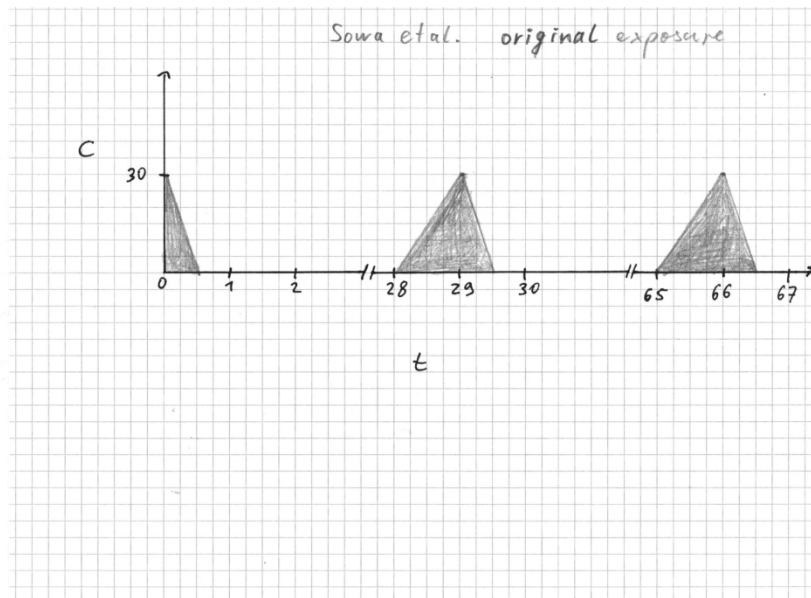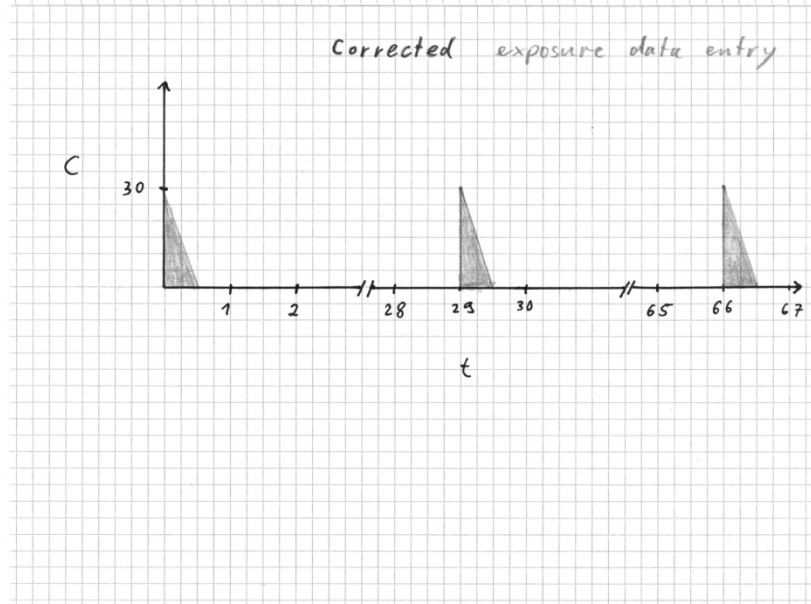

203x290mm (300 x 300 DPI)

Supplement: Supplementary file 1 — es4c03056_si_001.pdf [file es4c03056_si_001.pdf]

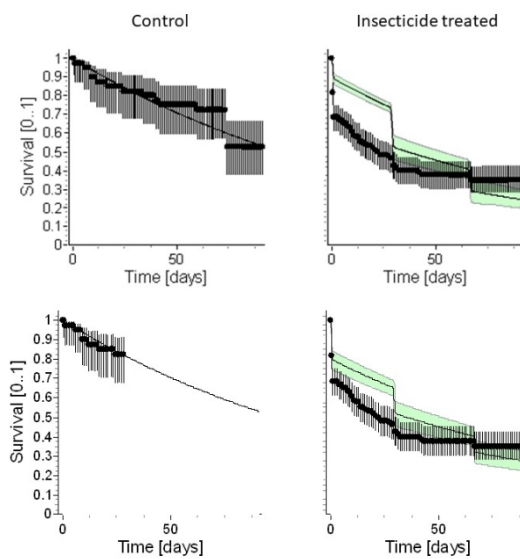

Original panels in Figure 6 of Sowa et al. 2024

Meadows – SD model

Corrected Figure

338x190mm (96 x 96 DPI)

Supplement: Supplementary file 2 — es4c03056_si_002.pdf [file es4c03056_si_002.pdf]
