# Supplementary material for: Correspondence on “Mortality Pattern of Poecilus cupreus Beetles after Repeated Topical Exposure to Insecticide—Stochastic Death or Individual Tolerance?”
Source: Environ Sci Technol. 2024 Jun 6;58(24):10874–6. doi: 10.1021/acs.est.4c03056 (PMC11191583; doi:10.1021/acs.est.4c03056)
Supplement: Supplementary file 4 — es4c03056_si_004.pdf [file es4c03056_si_004.pdf]

openGUTS Report

Project:

GUTS_OSR-all-guts-corrected-v2

Project file:

…\My results\GUTS_OSR-all-guts-corrected_v2.ogp

Project description (optional):

OSR: Data recalculated to correct for the number of beetles moved to acetone control after the 2nd and 3rd dosing to enable GUTS estimation throughout the whole experiment.

Software version:

openGUTS - 1.1

Date of report creation:

15/03/2024 08:56:53

Calibration

Calibration input data

Data set 1

File:

Description (optional):

OSR: Data recalculated to correct for the number of beetles moved

to acetone control after the 2nd and 3rd dosing to enable GUTS

estimation throughout the whole experiment.

Control group: 'OSR-A-corrrected'

Survival data of input data set 1:

Time [d]	OSR-A-corrrected	OSR-P-corrected
0	40	160
0.5	40	148
1	40	140
2	40	140
3	40	138
4	40	138
5	40	138
6	40	136
7	40	136
8	40	134
9	40	134
10	40	133
11	38	132
12	37	131
13	37	131
14	37	131
15	37	131
16	37	130
17	37	130

18	37	127
19	37	124
20	37	121
21	36	121
22	36	120
23	36	117
24	36	117
25	36	117
26	35	117
27	35	115
28	35	115
29	-1	115
29.5	-1	101
30	-1	101
31	-1	101
32	-1	101
33	-1	101
34	-1	97
35	-1	93
36	-1	91
37	-1	89
38	-1	89
39	-1	89
40	-1	85
41	-1	85
42	-1	85
43	-1	85
44	-1	85
45	-1	83
46	-1	83
47	-1	83
48	-1	81

49	-1	79
50	-1	79
51	-1	79
52	-1	77
53	-1	77
54	-1	77
55	-1	77
56	-1	77
57	-1	77
58	-1	77
59	-1	77
60	-1	77
61	-1	77
62	-1	77
63	-1	77
64	-1	77
65	-1	77
66	-1	77
66.5	-1	68
67	-1	68
68	-1	68
69	-1	68
70	-1	68
71	-1	68
72	-1	68
73	-1	68
74	-1	68
75	-1	68
76	-1	68
77	-1	68
78	-1	68
79	-1	68

80	-1	68
81	-1	68
82	-1	68
83	-1	68
84	-1	68
85	-1	68
86	-1	68
87	-1	68
88	-1	68
89	-1	68
90	-1	68
91	-1	68

Concentration data of input data set 1:

Time [d]	OSR-A-corrrected	OSR-P-corrected
0	0	30
0.5	0	0
29	0	0
29	0	30
29.5	0	0
66	0	0
66	0	30
66.5	0	0
91	0	0

Calibration settings

Calibration parameter settings for GUTS-RED-SD:

Parameter	Fit	Min	Max	Scale
kd	Yes	0.0005637	143.8	Log
mw	Yes	2.323E-5	29.7	Norm
hb	No	0.004725	0.004725	Norm
bw	Yes	3.859E-5	33200	Log
Fs	No	1	1	Norm

Calibration parameter settings for GUTS-RED-IT:

Parameter	Fit	Min	Max	Scale
kd	Yes	0.0005637	0.193	Log
mw	Yes	2.323E-5	1.617	Log
hb	No	0.004725	0.004725	Norm
bw	No	Inf	Inf	Norm
Fs	Yes	1.05	20	Log

Note:

Background hazard (hb) was prefitted to control.

Calibration results

Fitted parameters for GUTS-RED-SD:

Best fit parameter values and their 95% CI

kd: 0.3909 (0.1994 - 9.689)

mw: 2.519 (2.323E-5* - 6.625)

bw: 13.87 (0.01247 - 199.2)

* edge of 95% parameter CI has run into a boundary
  (this may also affect CIs of other parameters)

Goodness of fit for calibration data (GUTS-RED-SD):

Model efficiency (NSE, r-square): 0.9085

Normalised root-means-square error (NRMSE): 12.37 %

Minus log-likelihood (MLL): 484.32

AIC: 974.65

Survival probability prediction error (SPPE) for each treatment:

Data set	Treatment	Value
1	OSR-A-corrrected	-0.11 %
1	OSR-P-corrected	-2.13 %

GUTS-RED-SD results table for LCx,t [%RFD], with 95% CI:

Time [d]	LC50	LC20	LC10
1	9.341 (8.237 - 67.78)	8.63 (8.018 - 21.88)	8.355 (5.65 - 11)
2	5.219 (4.461 - 30.82)	4.96 (4.373 - 10.03)	4.858 (2.591 - 7.844)
3	3.975 (3.238 - 19.94)	3.828 (3.178 - 7.894)	3.77 (1.679 - 7.263)
4	3.405 (2.637 - 14.74)	3.307 (2.594 - 7.439)	3.268 (1.242 - 7.041)
7	2.79 (1.906 - 8.37)	2.746 (1.478 - 7.024)	2.729 (0.6977 - 6.817)
14	2.556 (1.514 - 7.166)	2.543 (0.7301 - 6.797)	2.539 (0.3448 - 6.702)
21	2.53 (1.438 - 6.971)	2.524 (0.4849 - 6.731)	2.523 (0.229 - 6.67)
28	2.524 (1.127 - 6.878)	2.521 (0.3629 - 6.7)	2.52 (0.1714 - 6.655)
42	2.521 (0.7501 - 6.786)	2.52 (0.2415 - 6.67)	2.52 (0.114 - 6.64)

50	2.521 (0.6297 - 6.758)	2.52 (0.2027 - 6.66)	2.519 (0.09574 - 6.636)
100	2.52 (0.3144 - 6.684)	2.519 (0.1012 - 6.636)	2.519 (0.0478 - 6.624)

Plots for GUTS-RED-SD calibration:

Parameter space plot for the calibration of GUTS-RED-SD:

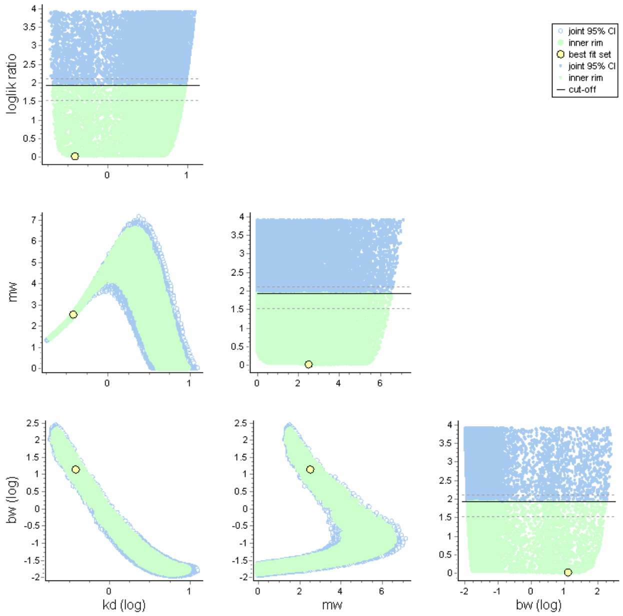

Exposure, damage and survival plots for the calibration of GUTS-RED-SD:

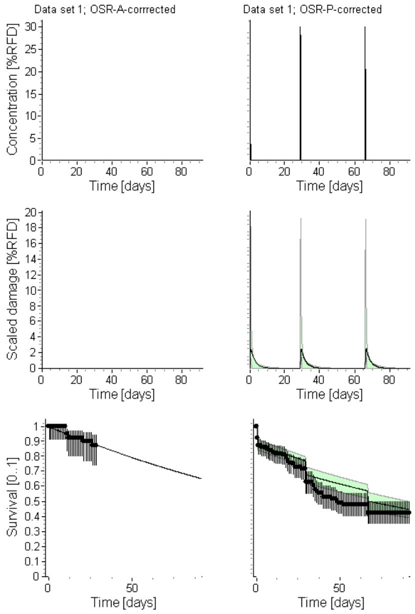

... continued plot:

Observed vs. Predicted survival plot for the calibration of GUTS-RED-SD:

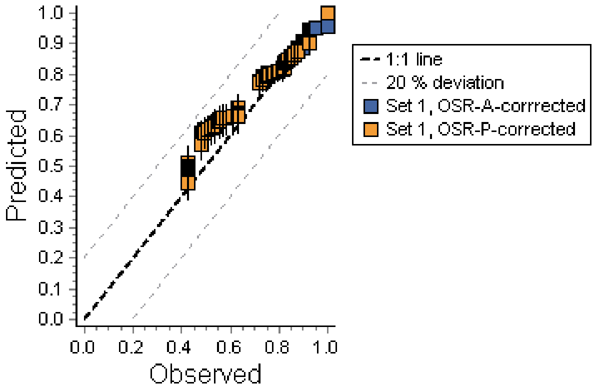

Observed vs. Predicted deaths plot for the calibration of GUTS-RED-SD:

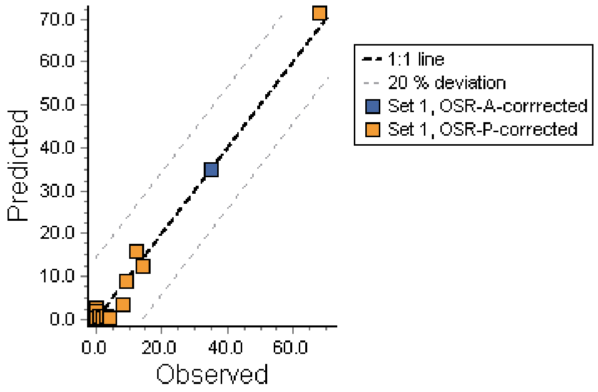

LCx versus time with confidence intervals (plotted for 16 days, GUTS-RED-SD):

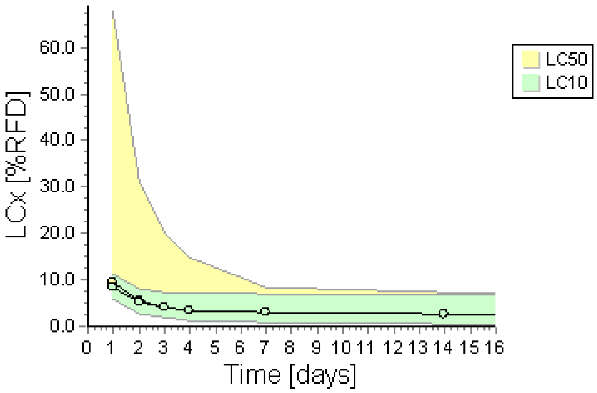

LCx [%RFD]
60.0
50.0
40.0
30.0
20.0
10.0
0.0
0 1 2 3 4 5 6 7 8 9 10 11 12 13 14 15 16
Time [days]
LC50
LC10

Fitted parameters for GUTS-RED-IT:

Best fit parameter values and their 95% CI

kd: 0.004696 (0.0005637* - 0.02241)

mw: 0.1601 (0.01707 - 0.4353)

Fs: 8.867 (2.742 - 20*)

* edge of 95% parameter CI has run into a boundary

(this may also affect CIs of other parameters)

Goodness of fit for calibration data (GUTS-RED-IT):

Model efficiency (NSE, r-square): 0.826

Normalised root-means-square error (NRMSE): 17.14 %

Minus log-likelihood (MLL): 495.13

AIC: 996.26

Survival probability prediction error (SPPE) for each treatment:

Data set	Treatment	Value
1	OSR-A-corrrected	-0.11 %
1	OSR-P-corrected	-4.5 %

GUTS-RED-IT results table for LCx,t [%RFD], with 95% CI:

Time [d]	LC50	LC20	LC10
1	34.17 (16.43 - 68.04)	14.96 (10.66 - 21.98)	9.23 (6.243 - 12.73)
2	17.12 (8.305 - 34.03)	7.498 (5.376 - 10.99)	4.626 (3.126 - 6.365)
3	11.44 (5.596 - 22.69)	5.011 (3.613 - 7.331)	3.091 (2.087 - 4.245)
4	8.602 (4.242 - 17.03)	3.767 (2.732 - 5.5)	2.324 (1.567 - 3.185)
7	4.95 (2.502 - 9.74)	2.168 (1.599 - 3.145)	1.337 (0.8995 - 1.822)
14	2.516 (1.345 - 4.882)	1.102 (0.8393 - 1.576)	0.6796 (0.4533 - 0.914)
21	1.705 (0.9625 - 3.263)	0.7464 (0.5787 - 1.054)	0.4604 (0.3037 - 0.6114)
28	1.299 (0.7675 - 2.453)	0.5689 (0.4422 - 0.7922)	0.3509 (0.2286 - 0.4607)
42	0.8943 (0.5715 - 1.643)	0.3916 (0.2964 - 0.5309)	0.2416 (0.153 - 0.3375)
50	0.7649 (0.5103 - 1.384)	0.3349 (0.2496 - 0.4473)	0.2066 (0.1288 - 0.3028)
100	0.4272 (0.307 - 0.7169)	0.187 (0.1266 - 0.2856)	0.1154 (0.0653 - 0.2238)

Plots for GUTS-RED-IT calibration:

Parameter space plot for the calibration of GUTS-RED-IT:

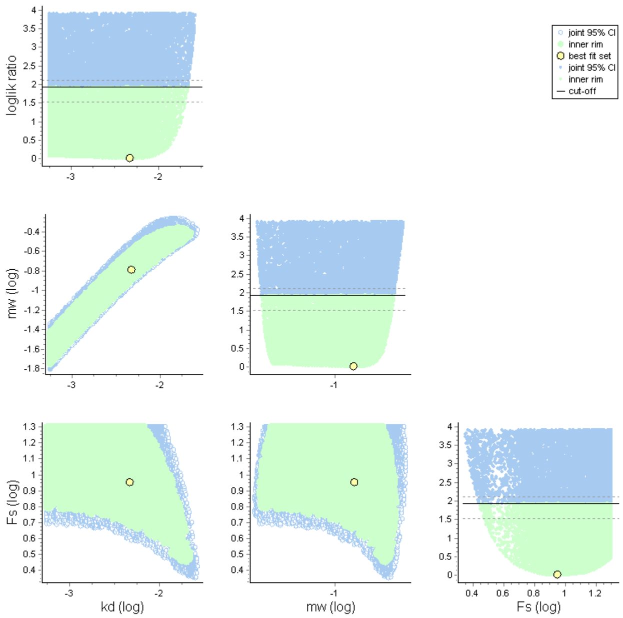

Exposure, damage and survival plots for the calibration of GUTS-RED-IT:

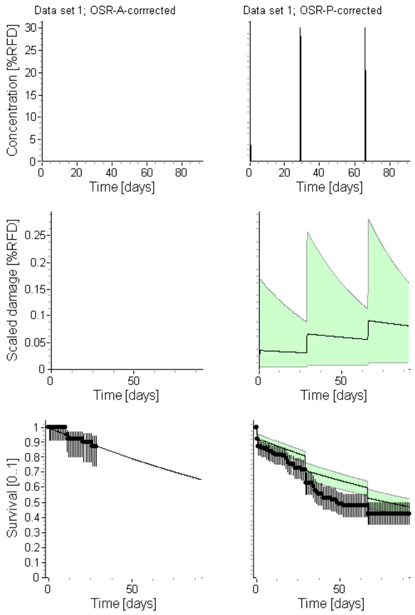

... continued plot:

Observed vs. Predicted survival plot for the calibration of GUTS-RED-IT:

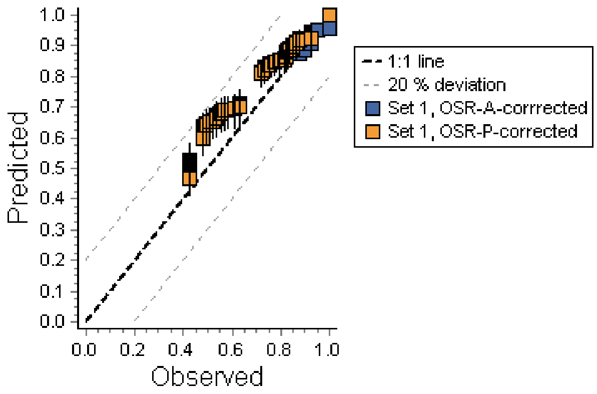

Observed vs. Predicted deaths plot for the calibration of GUTS-RED-IT:

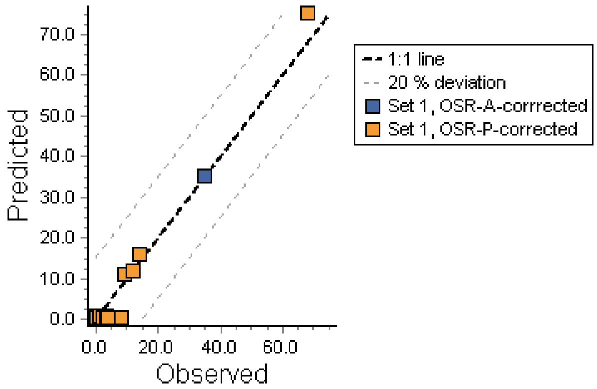

LCx versus time with confidence intervals (plotted for 16 days, GUTS-RED-IT):

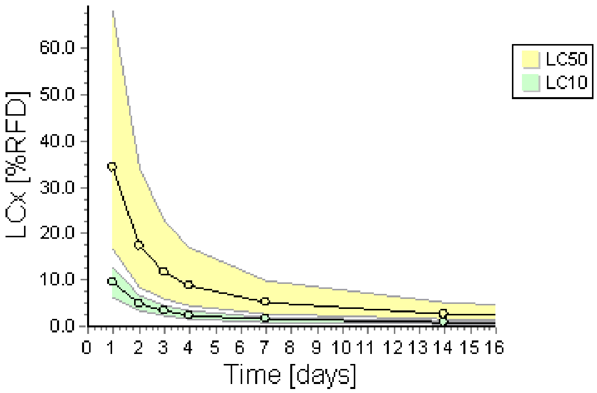

LCx [%RFD]
60.0
50.0
40.0
30.0
20.0
10.0
0.0
0 1 2 3 4 5 6 7 8 9 10 11 12 13 14 15 16
Time [days]
LC50
LC10

Validation

No validation performed!

Predictions

No predictions performed!
